# Efficacy and Durability of the Association of Botox and Skinvive in the Treatment of Moderate/Severe Wrinkles in the Periorbital Region: A Randomized, Controlled, Double‐Blind, Split‐Face Clinical Study

**DOI:** 10.1111/jocd.70403

**Published:** 2025-08-15

**Authors:** Maria Luiza Boechat Borges Neves, Cecilia Thome, Silvio Ventura da Silva Junior, Thalita Machado, Alfonso Sánchez‐Ayala, Mariana Barbosa Câmara‐Souza, Giancarlo De la Torre Canales

**Affiliations:** ^1^ Department of Dentistry Ingá University Center Paraná Brazil; ^2^ Private Practice Paraná Brazil; ^3^ Department of Dentistry University of Ponta Grossa Paraná Brazil; ^4^ Egas Moniz Center for Interdisciplinary Research (CiiEM) Egas Moniz School of Health and Science Almada Portugal; ^5^ Division of Oral Rehabilitation, Department of Dental Medicine Karolinska Institutet Huddinge Sweden

**Keywords:** botulinum toxin type a, Canthal wrinkles, hyaluronic acid filler, onabotulinumtoxinA, VYC‐12

## Abstract

**Background:**

The combined efficacy and durability of botulinum toxin A (BTX) and hyaluronic acid fillers (HA) for canthal wrinkles have not been thoroughly evaluated in randomized clinical trials.

**Aims:**

This study evaluated the efficacy, safety, and durability of the combined treatment protocol of onabotulinumtoxinA and Juvederm Volite VYC‐12 (Skinvive) for moderate and severe canthal lines.

**Methods:**

This randomized double‐blinded split‐face trial included 25 women with periorbital dynamic wrinkles at severity levels II–IV according to the Merz 5‐point scale. After bilateral onabotulinummtoxinA injections, the sides of the participants' faces were randomized to receive HA injections or a simulated injection. Assessed variables included electromyography activity (EMG), Merz 5‐point crow's feet scale, and FACE‐Q appraisal on crow's feet lines. Assessments were performed at baseline, 1, 2, 3, and 6 months. For differences in EMG and satisfaction scores, the two‐way repeated measures ANOVA and Bonferroni's post hoc analyses were conducted. Wrinkle severity scores were analyzed with the Mann–Whitney U test.

**Results:**

Inter‐treatment comparisons revealed no significant differences between treatment protocols in all assessed periods for EMG (*p* = 0.86). For severity of canthal wrinkles, onabotulinumtoxinA + VYC‐12 exhibited lower canthal wrinkle severity in rest and contracted positions after 3 (*p* = 0.04), and 3 (*p* = 0.007) and 6 (*p* = 0.001) months, respectively. Additionally, satisfaction with BTX‐HA treatment was significantly higher after 3‐month (*p* = 0.001) and 6‐month (*p* = 0.001) follow‐ups compared to BTX treatment.

**Conclusions:**

Higher improvements in canthal wrinkles could be enhanced by using onabotulinumtoxinA and VYC‐12 in association as a treatment protocol.

## 1 Introduction

The periorbital region is a critically important area of the face in terms of aesthetics, as it reflects health, vitality, and youthfulness when it appears luminous and rejuvenated [1]. Key characteristics of this region include well‐defined eyebrows, fullness in the upper and lower periorbita, distinct and clear eyelid folds, minimal excess skin, high‐quality skin, and the absence of canthal lines [2]. Nonetheless, the periorbital area is typically among the first facial regions to exhibit signs of aging [1]. The process of periorbital aging is complex and involves changes across all facial layers, from the bone to the skin [3]. Structural alterations, such as changes in the orbital septum, herniation of orbital fat, and a reduction in subcutaneous collagen, contribute to aging signs, including periorbital skin laxity, eyelid bags, and wrinkles [1, 4]. Furthermore, given that humans blink over 10 000 times daily, the repetitive contraction of the orbicularis oculi muscle significantly contributes to the development of wrinkles radiating from the outer corner of the eye to the temporal region, commonly referred to as “crow's feet” [1].

Due to the intricate nature of the aging process around the eyes, treating periorbital aging presents significant challenges. Currently, there is a wide array of minimally invasive antiaging treatments available for the periorbital region, with botulinum toxin type A (BTX) and hyaluronic acid (HA) fillers being among the most widely utilized. In addition, the 2023 International Society of Aesthetic Plastic Surgery (ISAPS) [5] international survey on aesthetic/cosmetic procedures identified BTX and HA as the most employed nonsurgical treatments globally.

Beyond its paralytic properties, BTX proves effective in addressing canthal dynamic wrinkles, irrespective of the brand used [6]. Therefore, despite the established efficacy of BTX in treating dynamic wrinkles, when these wrinkles become severe and manifest as static wrinkles, a combination of treatments may be required. In this context, HA injections can be particularly advantageous, as they not only restore volume but also enhance skin quality [7]. Given that the periorbital region undergoes constant movement, specific low‐crosslinking HA products, such as Juvederm VYC‐12 (currently marketed as Skinvive), should be prioritized [8, 9, 10]. Additionally, Juvederm VYC‐12 is recommended for treating fine cutaneous lines, and its beneficial effects on skin quality have been documented previously [9, 10, 11].

In light of the fact that both treatments are indicated for canthal lines and operate at different anatomical layers, it is plausible to suggest that their combined application may represent a promising strategy for addressing moderate to severe canthal wrinkles, particularly in cases where the efficacy of BTX monotherapy is insufficient. The synergistic potential of these treatments, when used in tandem, could enhance their clinical outcomes. However, the combined efficacy and durability of these treatments have not been thoroughly evaluated in randomized clinical trials. Consequently, the objective of this study was to evaluate the efficacy and durability of the combined treatment protocol of onabotulinumtoxinA and Juvederm Volite VYC‐12 (Skinvive) for moderate and severe canthal lines.

## 2 Materials and Methods

This study was a randomized and double‐blinded clinical trial approved by the Research Ethics Committee of Uningá University (CAAE: 77000724.0.0000.5220). The study was conducted at a specialized aesthetic clinic between April and December 2024. Participants were requested to give their signed written consent to join the study. The study adhered to the Helsinki Declaration and followed the CONSORT checklist.

### 2.1 Participants

The study sample consisted of Brazilian women aged 35–70 years, who expressed concerns about dynamic wrinkles around the eyes (periorbital wrinkles) at severity levels II–IV according to the Merz 5‐point scale [12]. Exclusion criteria ruled out patients who had undergone BTX injections for cosmetic or therapeutic purposes, as well as any cosmetic procedures (surgical and non‐surgical procedures) in the upper third of the face within the 12 months prior to the study. Additionally, individuals with autoimmune and neuromuscular disorders, those who had received any type of vaccine in the 3 months before starting the study, and those taking medications that could impact neuromuscular junctions were also excluded.

The sample size calculation was performed using G*Power 3.1.9.2 software (version 3.1.9.2, Kiel, Germany), based on a pilot study including three participants (*n* = 3) in each group, focusing on the electromyography of the orbicularis oculi muscle. The following parameters were considered for an ANOVA with repeated measures and within‐between interaction: *α* error probability = 0.05, power (1—*β* error probability) = 0.8, number of groups = 2, number of measurements = 5, and effect size *f* value = 0.427. The total sample size calculated was 40 face sides (24 subjects). Accounting for an anticipated withdrawal or dropout rate of 20%, a total of 50 face sides (25 subjects) were included to be distributed among the groups.

### 2.2 Study Protocol

Throughout the study, participants completed six evaluations. During the initial visit, participants were screened based on the outlined criteria. During the second visit, baseline variables were measured, and BTX injections were performed. Subsequent follow‐up evaluations were conducted 1 month later (Visit 3), wherein the sides of the participants' faces were randomized: one side received HA injections while the contralateral side underwent a simulated injection (cannula insertion and technique injection without gel administration). Further assessments were performed 2‐, 3‐, and 6‐month post‐BTX treatment.

### 2.3 Randomization and Blinding

An online computer program (http://www.randomization.com/) was used to randomize participants in blocks of two, with the process overseen by a technician not involved in any other part of the study. For each participant, the technician prepared a note specifying which site of the face would receive the HA injections and placed it into a sealed, opaque envelope. The randomization list was kept hidden from the researcher examining the patients (M.L.B.B.N.) and the participants themselves until the data collection phase was completed. An experienced specialist in aesthetic procedures performed the aesthetic injections and was not involved in any other aspects of the study.

### 2.4 Interventions

BoNT‐A injection protocols were performed according to the published consensus for onabotulinumtoxinA [13]. The injection protocol involved reconstituting BoNT‐A vials (Botox/onabotulinumtoxinA, 200 U AbbVie, USA) with 2 mL of 0.9% isotonic sterile saline solution, stored at room temperature, giving a dose of 10 U/0.1 mL. The total BoNT‐A doses administered in the orbicularis oculi muscles were 36 U per patient, divided into three injection points per muscle (6 U each/18 U total). Briefly, injections in the orbicularis oculi muscles were given in a medial‐to‐lateral direction before the peak of zygomatic bone protrusion. No additional injection points were used throughout the study. Injections were done using 31G × 6 mm needles.

For HA injections in the periorbital region, VYC‐12 Juvederm Volite, now marketed as Skinvive (AbbVie, USA), was used. Injections totaling 0.5 mL per site were administered into the superficial subcutaneous tissue using a retrolinear technique, following the direction of the lines. On the contralateral site, a simulated injection was performed by inserting a cannula and employing the same retrolinear technique; however, no HA was injected. Great care was taken to ensure that the patients did not notice whether the syringe was empty or not. Injections and simulations of injections were done using a 22G × 50 mm cannula.

### 2.5 Outcomes

Patient outcomes were assessed at five intervals: baseline, and then at 1, 2, 3, and 6 months. The researcher (M.L.B.B.N.) conducting measurements did not participate in any other part of the study. The primary outcome of the study was the electrical activity of the muscles.

#### 2.5.1 Electromyography (EMG)

To record the electromyographic (EMG) signals, a four‐channel EMG system (Miotool NG USB, Porto Alegre, RS, Brazil, frequency range: 10–700 Hz; sampling rate: 3000/s; resolution: 2.44 V/bit) was employed by a single, trained operator. Bipolar surface electrodes measuring 3.2 × 2.8 cm (Ag‐AgCl disks, Covidien LLC, Quebec, Canada) were applied to the target muscles after cleaning the skin with 70% alcohol [14]. The reference electrode was positioned on the manubrium of the sternum. The electrical activity of the orbicularis oculi muscle was recorded during maximum voluntary contraction (MVC). For this, patients were instructed to contract the muscle by closing their eyes and smiling simultaneously. To capture the EMG signal during MVC, participants were instructed to contract their designated muscles as forcefully as possible for 5 s. This process was repeated three times, allowing for a 2‐min rest interval between each measurement to prevent muscle fatigue. The EMG signals were recorded at a frequency of 1000 Hz and subsequently band‐pass filtered between 20 and 500 Hz to determine the root‐mean‐square (RMS). For data analysis, MiotecSuite software version 1.0 (Miotec Equipamentos Biomédicos, Porto Alegre, Brazil) was utilized, and the average of the three MVC recordings was used for statistical evaluation.

#### 2.5.2 Severity of Canthal Lines

The severity of canthal lines was evaluated using the Merz 5‐point crow's feet grading scale to rate the visible lines of orbicularis oculi muscles. This method rates the appearance of lines in both their most contracted and relaxed states as follows: 0 indicates no lines, 1 signifies mild lines, 2 represents moderate lines, 3 corresponds to severe lines, and 4 denotes very severe lines [12]. The patients rated the severity of canthal lines based on a visual examination.

#### 2.5.3 Patient's Perceived Satisfaction With BoNT‐A Treatment

The FACE‐Q scales are established tools designed to measure both expectations and satisfaction of patients before and after undergoing treatment. In this study, the FACE‐Q assessment specific to crow's feet lines was used to gauge patient satisfaction with their treatment. This particular scale measures how much their crow's feet lines (the lines at the outer corners of the eyes) have bothered participants in the past week, utilizing a 4‐point scale: 1 means “not at all,” 2 signifies “a little,” 3 stands for “moderately,” and 4 indicates “extremely.” Scoring is conducted by adding up the scores from each individual item to produce a total raw score. This raw score is then transformed, using a specific conversion table, into a scale ranging from 0 (indicating the poorest possible outcome) to 100 (reflecting the best possible outcome) [15, 16].

The authors of this study have secured a license agreement to employ the FACE‐Q scales for nonprofit academic research purposes.

### 2.6 Statistical Analysis

The data were analyzed using IBM SPSS Statistics (version 25, IBM: NYSE, Armonk, NY, USA). All inferences were conducted with two‐tailed tests, assuming a significance level of 95% (Type I error, *α* = 0.05) and a statistical power of 80% (Type II error, *β* = 0.2). The assumptions of normality and homogeneity of variances were verified using the Shapiro–Wilk test and Levene's test, respectively. Differences in electromyography (EMG) activity and satisfaction scores based on time and treatment were analyzed using a two‐way repeated measures ANOVA. Post hoc analysis was conducted with Bonferroni's correction for marginal means comparisons. The assumption of sphericity was verified using Mauchly's test of sphericity. Meanwhile, wrinkle severity scores were analyzed with the Mann–Whitney U test.

## 3 Results

### 3.1 Study Population

**FIGURE 1:**
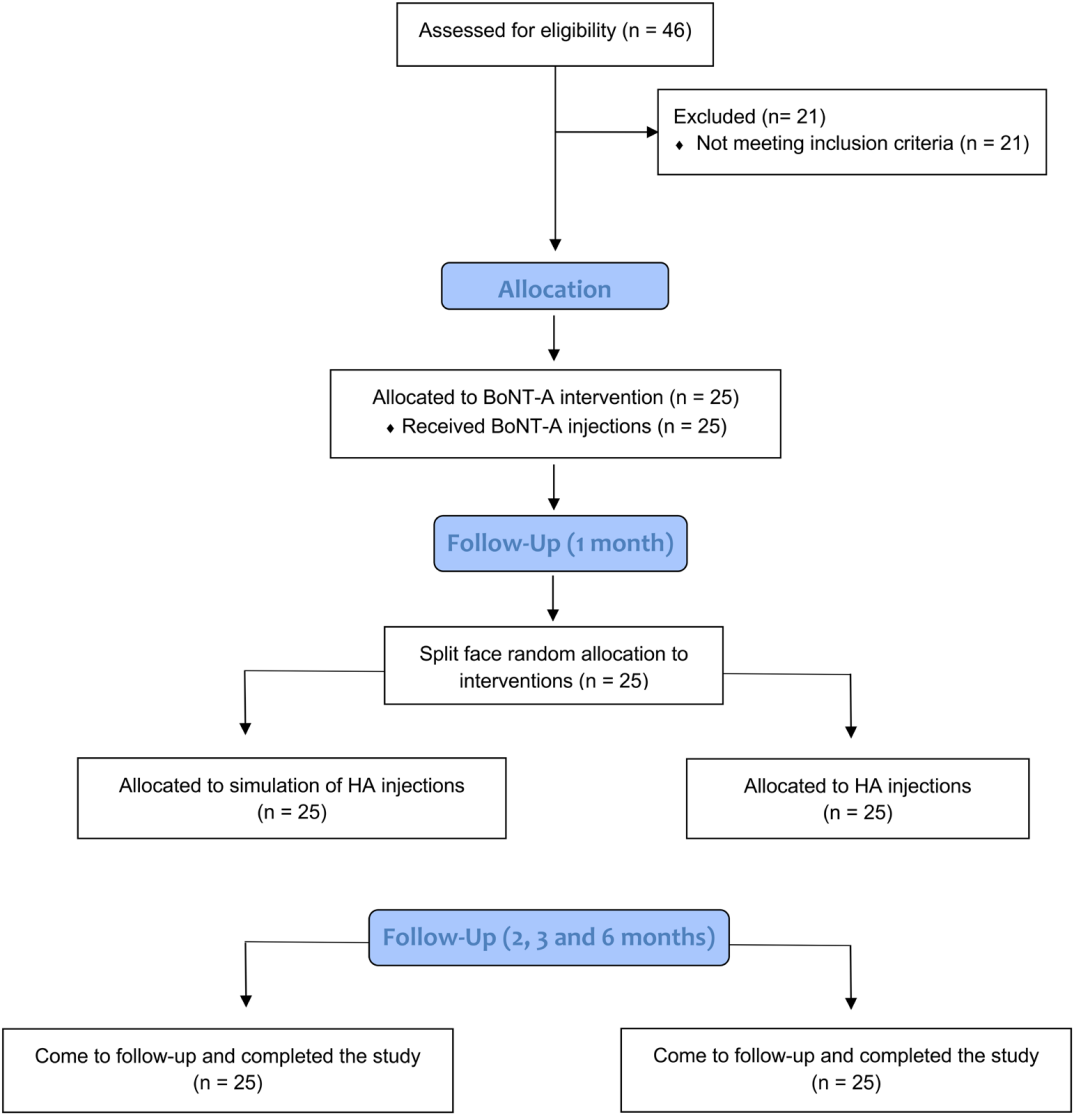
CONSORT flow diagram.

In total, 46 Brazilian women patients underwent screening; 21 did not meet the inclusion criteria, leading to the enrolment of 25 patients (48.8 ± 8.3) in this trial. No dropouts were registered through the 6‐month study period (Figure 1). Furthermore, 14 (56%), 5 (20%), and 6 (24%) patients were classified as presenting moderate, severe, and very severe canthal lines, respectively.

### 3.2 Electromyographic Activity

**FIGURE 2:**
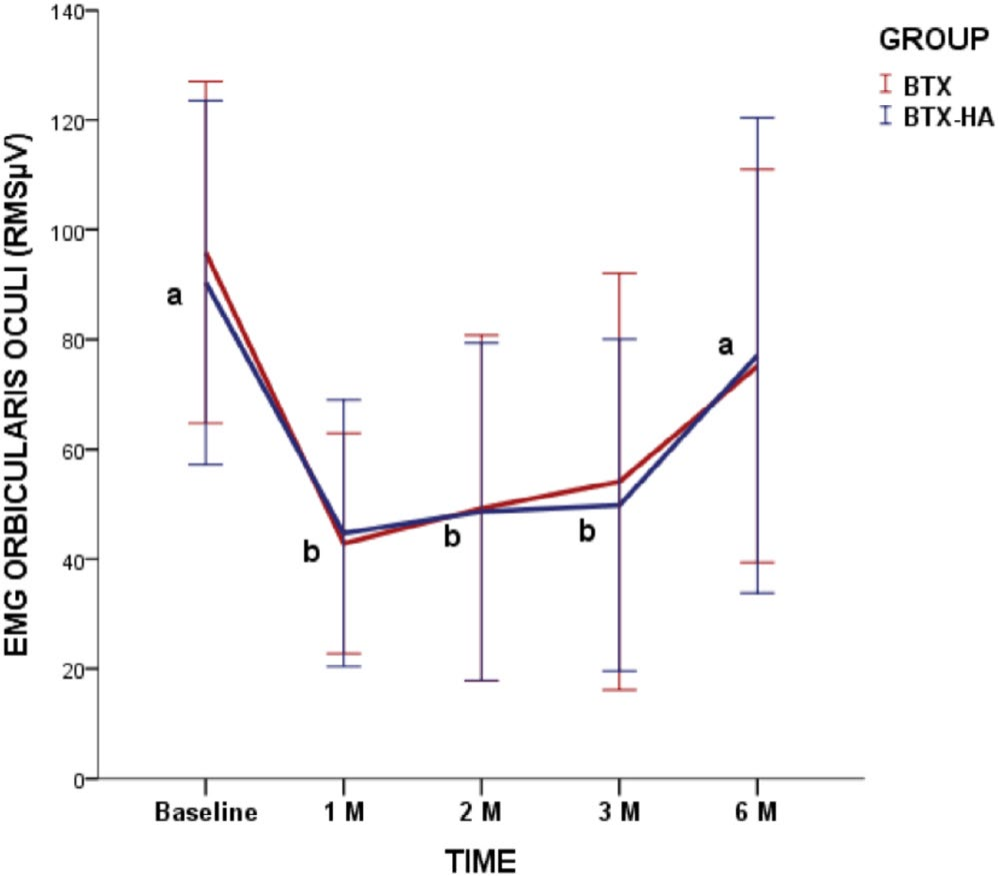
Changes in root‐mean‐square scores (RMS μV) in maximum voluntary contraction (MVC) of orbicularis oculi according to treatments and timepoints. Different lowercase letters mean intragroup significant difference at 0.05. No intergroup differences were found in the study.

Intra‐treatment comparisons revealed significant reductions in the electrical activity of the orbicularis oculi muscle at the 1‐, 2‐, and 3‐month follow‐ups for both treatment protocols compared to baseline (*p* = 0.0001). Inter‐treatment comparisons revealed no significant differences between treatments in all assessed periods (*p* = 0.86) (Figure 2).

### 3.3 Canthal Lines Severity

**FIGURE 3:**
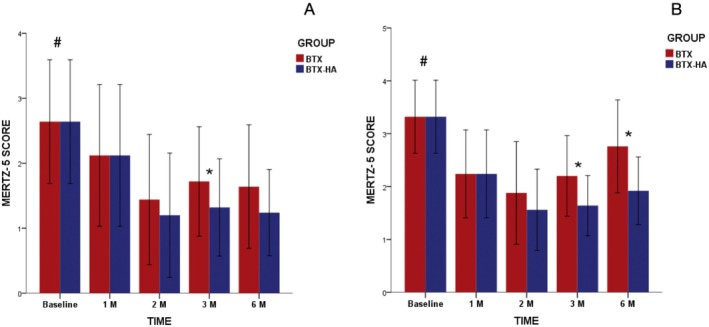
Severity of canthal lines (Merz scale scores) according to treatment and timepoints at (A) rest and (B) contracted positions. ^#^Intragroup significant differences at 0.05. *Intergroup significant differences (*p* < 0.05).

Intra‐treatment comparisons showed significant reductions in canthal wrinkles severity for both treatment protocols across all follow‐ups when compared to baseline in rest (*p* = 0.04) (Figure 3A) and contracted (*p* = 0.01) (Figure 3B) positions. Inter‐treatment comparisons in rest position exhibited lower canthal wrinkles severity for BTX + HA compared with BTX just at the 3‐month assessment (*p* = 0.04). Regarding the contracted position, higher reductions of canthal wrinkles severity were found in the BTX + HA treatment than in BTX when compared at 3‐month (*p* = 0.007) and 6‐month (*p* = 0.001) follow‐ups.

### 3.4 Patient Satisfaction With Treatment

**FIGURE 4:**
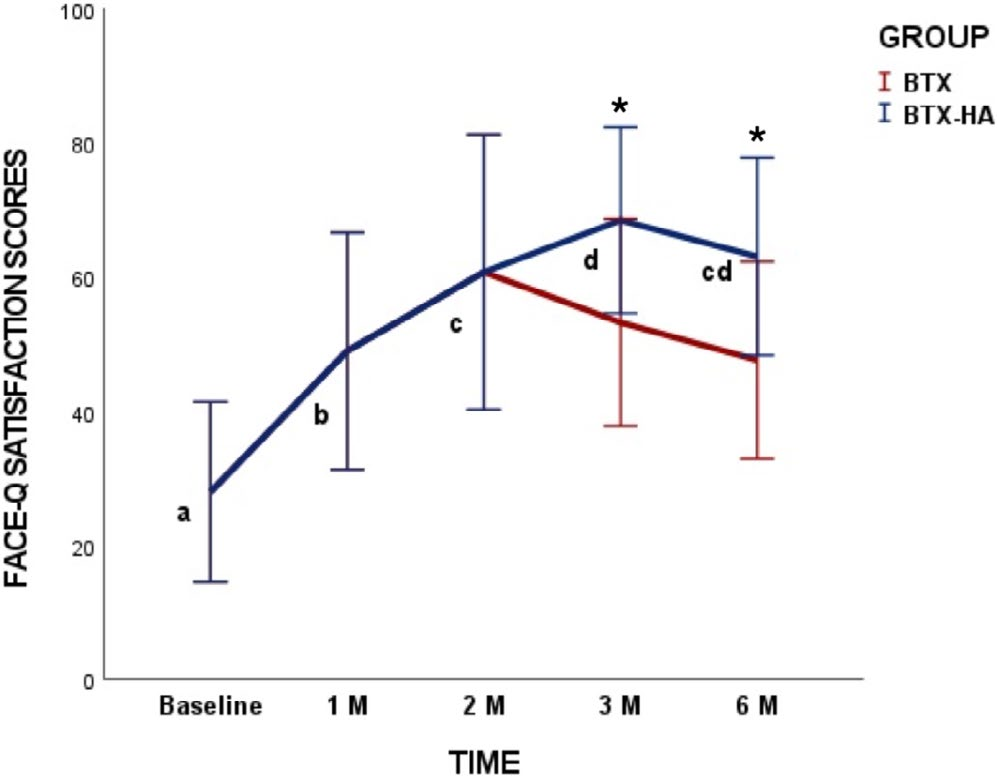
Face‐Q treatment satisfaction scores according to treatment and timepoints. Different lowercase letters mean significant differences at 0.05. *Intergroup significant differences (*p* < 0.05).

Regarding intra‐treatment comparisons, both treatment protocols improved patient's satisfaction with treatment at 1‐, 2‐, 3‐, and 6‐month follow‐ups compared with baseline (*p* = 0.0001). Inter‐treatment comparisons showed that satisfaction with BTX‐HA treatment was significantly higher after 3‐month (*p* = 0.001) and 6‐month (*p* = 0.001) follow‐ups compared to BTX treatment (Figure 4).

### 3.5 Adverse Events

No adverse events were reported for any of the participants in both groups throughout the study.

## 4 Discussion

Even though the effects of BTX and HA on wrinkles are worldwide known and the association of these treatments is clinically common, as far as the authors know, this is the first study in assessing the efficacy of the association of these therapies as treatment for canthal wrinkles. The main findings of our study show that when onabotulinumtoxinA and VYC‐12 are used in tandem, a higher efficacy and longer term effects are reached regarding the diminution of canthal wrinkles severity and patients' satisfaction with treatment. Additionally, this association of treatments is safe since no adverse effects were reported.

Regarding canthal wrinkles severity results, our study demonstrated that onabotulinumtoxinA treatment was efficient in reducing the EMG activity of the orbicularis oculi muscle until the 3‐month assessment, and consequently improving the perception of the patients related to the severity of canthal wrinkles, as shown by the scores of the Merz 5‐point crow's feet scale. These findings align with plenty of studies using the same BTX brand [17, 18, 19]. It is important to mention that besides the subjective assessment used by the mentioned studies, our study also used an objective assessment (EMG) which strengthens our results. Interestingly, it seems that the effectiveness of BTX for canthal lines is independent of the brand used, since other studies using abobotulinumtoxinA [6] and RelabotulinumtoxinA [20] rely on the same results.

Interestingly, even though we did not find a significant reduction in the EMG activity of the orbicularis oculi muscle at the 6‐month follow‐up, the perception of the patients regarding the severity of canthal wrinkles in the face sites that were treated with the association of onabotulinumtoxinA and VYC‐12 was still improved at this follow‐up. The active injections of VYC‐12 could be the main explanation for this sustained improvement. Juvederm Volite VYC‐12 (Skinvive) is an injectable crosslinked HA developed using Vycross technology indicated for fine lines and to improve skin quality attributes such as hydration and elasticity [21]. Therefore, it is quite possible that the sustained improvement in canthal wrinkles shown in our study after treatment injections is more related to the improvement of skin appearance, which consequently influences the appearance of wrinkles. The positive effects of VYC‐12 on skin quality have already been proven in other parts of the face by other studies [10, 22, 23]. In addition, a retrospective case review demonstrated the safety of VYC‐12 as a combination treatment (neuromodulators and fillers) to improve skin quality and hydration [24]. Also, a prospective study reported VYC‐12 positive effects in reducing horizontal line severity in the neck, with progressive improvements with more treatment injections [25].

Furthermore, the satisfaction with the treatment of canthal wrinkles was significantly higher after the injection of VYC‐12. This could be related to the fact that we used two injectable substances (onabotulinumtoxinA + VYC‐12) that present different mechanisms of action and that are injected in different layers (muscle and skin), making a more complete treatment for this area. Additionally, this higher satisfaction could also be related to the fact that even though the severity of canthal wrinkles was improved, these wrinkles were not eliminated, which is in line with the natural look that the patients request nowadays in clinical practice [26]. In addition, these lines are associated with mimics that correspond to positive emotions; consequently, maintaining these lines in low severity could hence enhance patients' well‐being. This is important given the influence of dynamic facial emotions on social perception [27, 28].

While the principal strength of this study is its robust methodological design (randomized, double‐blinded, and split‐face design), certain limitations should be mentioned. The exclusive inclusion of female patients limits the generalization of the results to male populations, since male participants could need different treatment doses to enhance the same results. Also, including objective and subjective assessment of skin quality is reasonable due to VYC‐12 properties and could bring new knowledge about the association of these treatments for canthal wrinkles. Furthermore, our study presented a combination of objective and subjective assessments and followed the consensus of each of the injectable treatments used. Additionally, the novelty of the findings contributes to the knowledge of aesthetic treatment for the periorbital region. We recommend that clinicians use this association of minimally invasive treatments in patients that present severe canthal wrinkles and skin damage. Future research should explore the efficacy of the association of other brands of BTX and HA to see if they are equally effective.

## 5 Conclusion

The study concludes that the association of onabotulinumtoxinA and Juvederm Volite VYC‐12 (Skinvive) produces positive and safe results for severe canthal wrinkles.

## Author Contributions

Conceptualization: M.L.B.B.N. and G.D.T.C.; data curation: M.L.B.B.N., T.M, and S.V.S.J.; formal analysis: G.D.T.C. and A.S.‐A.; investigation: M.L.B.B.N., C.T, and S.V.S.J.; methodology: M.L.B.B.N., A.S.‐A., M.B.C.‐S., and G.D.T.C.; project administration: G.D.T.C. and M.B.C.‐S.; writing – original draft: M.L.B.B.N., G.D.T.C., and A.S.‐A.; writing – review and editing: M.L.B.B.N., M.B.C.‐S. and G.D.T.C. All authors have read and agreed with the final version of the manuscript.

## Ethics Statement

This research received approval from the Research Ethics Committee of Uningá University (CAAE: 77000724.0.0000.5220). All participants were asked to provide a signed written consent to take participate in the study.

## Conflicts of Interest

The author Maria Luiza Boechat Borges Neves works as a speaker for Abbvie—Allergan, Brazil. All other authors declare no conflicts of interest.

## Data Availability

All data are available upon reasonable request to the corresponding author.
